# Sperm quality metrics were improved by a biomimetic microfluidic selection platform compared to swim-up methods

**DOI:** 10.1038/s41378-023-00501-7

**Published:** 2023-03-28

**Authors:** Steven A. Vasilescu, Lin Ding, Farin Yazdan Parast, Reza Nosrati, Majid Ebrahimi Warkiani

**Affiliations:** 1 NeoGenix Biosciences pty ltd, Sydney, NSW 2126 Australia; 2 School of Biomedical Engineering, University of Technology Sydney, Sydney, NSW 2007 Australia; 3 Department of Mechanical & Aerospace Engineering, Monash University, Clayton, VIC 3800 Australia; 4 Institute for Biomedical Materials & Devices (IBMD), Faculty of Science, University of Technology Sydney, Sydney, NSW 2007 Australia

**Keywords:** Materials science, Engineering

## Abstract

Sperm selection is an essential component of all assisted reproductive treatments (ARTs) and is by far the most neglected step in the ART workflow in regard to technological innovation. Conventional sperm selection methodologies typically produce a higher total number of sperm with variable motilities, morphologies, and levels of DNA integrity. Gold-standard techniques, including density gradient centrifugation (DGC) and swim-up (SU), have been shown to induce DNA fragmentation through introducing reactive oxygen species (ROS) during centrifugation. Here, we demonstrate a 3D printed, biologically inspired microfluidic sperm selection device (MSSP) that utilizes multiple methods to simulate a sperms journey toward selection. Sperm are first selected based on their motility and boundary-following behavior and then on their expression of apoptotic markers, yielding over 68% more motile sperm than that of previously reported methods with a lower incidence of DNA fragmentation and apoptosis. Sperm from the MSSP also demonstrated higher motile sperm recovery after cryopreservation than that of SU or neat semen. Experiments were conducted side-by-side against conventional SU methods using human semen (*n* = 33) and showed over an 85% improvement in DNA integrity with an average 90% reduction in sperm apoptosis. These results that the platform is easy-to-use for sperm selection and mimics the biological function of the female reproductive tract during conception.

## Introduction

Infertility is a growing global health issue with significant psychological, social, and economic implications, affecting over 185 million individuals worldwide^1^. In Australia, 1 out of every 6 couples experience infertility issues, and 1 in every 22 children is now born via assisted reproduction. Male infertility solely contributes to ~30% of infertility cases globally^2^ and to 40% of infertility cases in Australia^2,3^. In recent trends, the first pregnancy is often postponed, demonstrating the limits of natural fertility and accelerating the need for medical intervention and innovation in ART to overcome these limits. Therefore, to treat the increasing demand for infertility treatment effectively, it is vital that the methods and technologies used in ART continue to improve, particularly when male factor infertility is concerned. Ensuring quality sperm selection is crucial to the success of assisted reproduction since it influences many factors that contribute to the success of assisted reproductive treatments (ARTs)^3–5^. Poor sperm quality correlates with an increased risk of birth defects, lower embryo fertilization rates, lower embryo quality, and lower implantation rates and has a negative association with live birth rates after in vitro fertilization (IVF)^6–9^.

The quality of selected sperm is also heavily reliant upon the skill of the embryologist and unstandardized preparation protocols, often resulting in operator-to-operator variations in IVF success^4^. This is most prominent in the sperm processing stage, which consists of gradient centrifugation, cell resuspension and delicate aliquoting^10^. The most common clinical methods of sperm selection are DGC and SU. These conventional techniques circumvent natural sperm filters, neglecting important factors, such as DNA integrity and sperm apoptosis. In fact, centrifugation-based selection approaches may induce sperm DNA fragmentation (sDF) in certain samples or will fail to reduce it^11–14^. Sperm cells in which the genetic material is significantly damaged, in the form of DNA fragmentation, have been shown to increase the risk of miscarriage up to 3.94 times^8,15^. In attempts to provide alternatives to conventional centrifugation-based methods of sperm selection, several groups have utilized microfluidics to perform sperm selection. Microfluidic devices have been developed to select sperm typically through motility-based behavioral mechanisms, thereby preventing the oxidative stress and DNA fragmentation induced by centrifugation^3,4,16^. The clinical translation of these technologies has been infrequent^17^, largely due to their complexity of operation. Without an intuitive user interface, many devices have not undergone side-by-side clinical testing to evaluate their performance by clinicians. A successful sperm selection platform must not only provide high-quality sperm in a timely manner but also be simple to use and consistent in its performance^3,18^. Many devices also rely on a single mechanism (typically motility) to select sperm, while those that do not, are overly complex and are often used to assess rather than recover sperm^19^. Motility-based sperm selection is inspired by the natural progression of sperm through the female reproductive tract (cervical crypts, uterine cavity, intratubal junction, fallopian tube, etc.), and there are a multitude of selective mechanisms that impact the migration of sperm before fertilization^5^. Natural sperm selection facilitates a reduction in sperm from 300 million upon ejaculation down to approximately 250^20^ through a variety of mechanisms. Therefore, using a combination of mechanisms for sperm selection may provide more fecund sperm, particularly for ICSI. One mechanism involves the tagging and removal of apoptotic sperm using Annexin V. The proportion of early apoptotic sperm cells in raw semen has been shown to reach over 20%^21^, and it has been reported that eliminating apoptotic sperm using annexins correlated with an improvement in embryo quality^22^. Annexin A5 (AAV) is an example of one such apoptotic marker that binds to phosphatidylserine externalized in apoptotic cells^22^. This action reduces the impact of cells undergoing both spontaneous and testicular-induced cell death via apoptosis during conception. Apoptosis is an essential mechanism that occurs in both fertile and (to a larger extent) infertile men to eliminate unwanted cells due to triggering stimuli (deprivation of intratesticular testosterone and gonadotrophins, Sertoli cell toxicants, chemotherapeutic drugs, and temperature imbalances)^23^. Apoptosis is important in the context of sperm due to the errors that occur in cells during their production and the resulting need for cell death to eliminate cells with genetic defects. The presence of apoptotic sperm in semen has been shown to be more prevalent in infertile men^24^, especially those with unidentified male infertility^25^. Apoptotic sperm also present more frequently with DNA fragmentation, which is a key performance indicator for sperm quality and future embryo quality^26^. Although technologies such as magnetic-activated cell sorting (MACS) are available for removing apoptotic sperm cells through AAV binding, they only exist as an adjunct to conventional DGC or SU techniques. MACS by itself has been shown to provide little benefit to the overall quality of sorted sperm populations but has proven effective when used post DGC or SU^27,28^. MACS-sorted sperm have been shown to exert a beneficial effect on pregnancy rate and sperm cryosurvival when compared to that of conventional methods^29^. However, using MACS dramatically compounds the amount of time, equipment and human intervention needed for sperm preparation, making the method less appealing to clinics. Therefore, combining the use of Annexins with motile sperm selection in a single platform could further improve sperm quality without reducing clinical efficiency.

Here, we report a hybrid microfluidic sperm selection platform (dubbed the hybrid MSSP) that can select sperm with considerably improved quality in 15 min, a quarter of the time needed for SU, significantly reducing the time on task for embryologists and the amount of time sperm must spend in vitro. The sperm can be selected via motility-based boundary following alone or in combination with apoptotic sperm trapping. 3D printing was used to maximize the yield of healthy sperm recovered from raw human semen by creating a pattern of layered ridges for sperm to follow, resulting in an average 68.4% concentration yield increase over previously reported straight channels^30^. Sperm populations isolated from the device demonstrated considerable improvements in motility (93.5% vs. 74.1%), vitality (97.6% vs. 86.0%), DNA integrity (1.4% vs. 7.9%), cryosurvival (64.2% vs. 52.8%), and apoptotic marker expression (5.66% vs. 26.5%) compared with a conventional SU-based approach. Furthermore, our microfluidic platform achieves these results with a higher degree of consistency and fewer steps, representing a clinically viable approach to sperm selection that may benefit the downstream process and overall success of ART.

## Materials and methods

### Device fabrication

Devices were fabricated using modified additive manufacturing techniques previously reported by our group for inertial microfluidic devices^31,32^. 3D printing was performed using a high-resolution Digital Light Processing (DLP) 3D printer (MiiCraft, Hsinchu Taiwan). The desired geometry was drawn in SolidWorks 2018 ×64 Premium Edition and then exported as an STL file to Miicraft software (MiiCraft 125, Version 4.01, MiiCraft Inc) for preprocessing of the printing options. The printer projects a 405 nm UV wavelength through the resin (BV-007, MiiCraft Inc.) to solidify the liquid photopolymer in a solid layered structure. Details of the resin structure have been previously reported, and their effects on sperm cell vitality have been investigated^32,33^. The microfluidic device used a circular array of 184 microchannels, each with a height of 600 µm and a length of 7.5 mm. Each pair of channels congregates into a single channel after 3 mm and includes a series of ridges along the entire length of the channel walls, which increases the number of surfaces and boundaries that can bear sperm while also allowing for an overall larger entry to each channel (Supplementary Fig. 2). As the sperm converge on the center of the chip at the end of the channels, a circular pattern of crescent moon-shaped pillars was situated to help retain sperm in the center of the chip and prevent them from exiting the collection area. After printing, the top half of the chip was thoroughly washed with isopropanol alcohol (IPA) and DI water (three times). Between each wash, the part was blow-dried with a pressurized air gun, ensuring that all residual liquid resin was removed while not damaging the structures. The part was then cured under ultraviolet (UV) light for 120 s. Once the chip was ready, it was attached to a poly-methyl methacrylate (PMMA) sheet using transparent double-sided pressure-sensitive adhesive tape (ARcare, Adhesive Research) coated with AS-110 acrylic medical grade adhesive. This approach effectively binds open 3D-printed microchannels with optically transparent acrylic sheets, producing a tightly sealed microchannel that allows on-chip microscopy.

### Semen preparation

Human semen samples were obtained through ejaculation after 2–7 days of sexual abstinence, as recommended by the World Health Organization (WHO)^10^. Raw semen samples (*n* = 33) were incubated at 37 °C for 20 min to achieve for full liquefaction. To simulate oligozoospermia, defined by the WHO as a sperm concentration of less than 15 million sperm per mL^1^, and compare selection methods on oligozoospermic samples, additional raw semen was diluted with Sperm Rinse media to concentrations below 15 million sperm per mL (*n* = 6). All donors signed an informed consent. This study was approved by the ethics review board at UTS (ETH19-3677).

### Device operation

#### Motility-based sperm selection

**Fig. 1:**
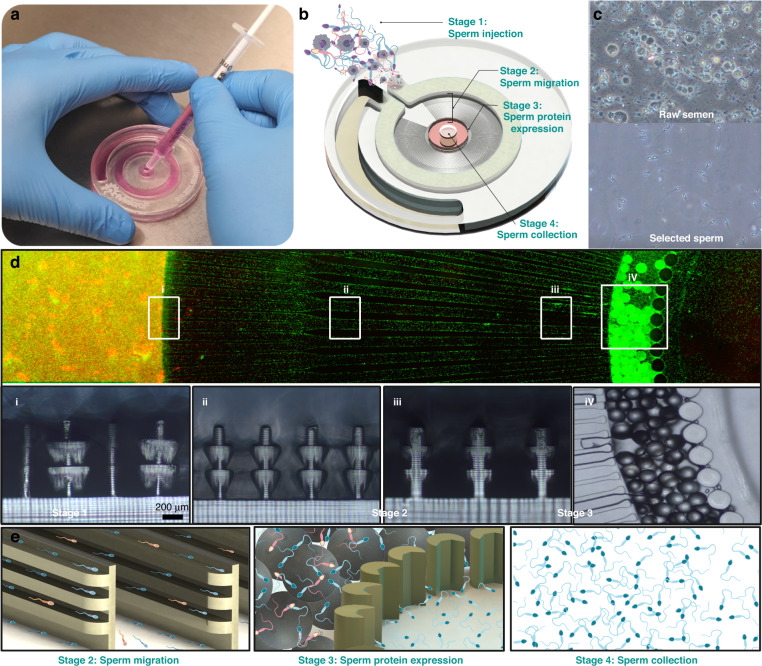
Overview of the sperm selection process within the microfluidic device and representative geometry. **a** Image of the device filled with food dye for visualization. **b** Schematic overview of the device showing each stage of operation after buffer loading, including stage 1—semen injection into the device, stage 2—sperm migration through layered 3D printed microchannels, stage 3 (optional hybrid version only)—annexin-positive sperm trapping, and stage 4—sperm collection from the center of the chip. **c** Images before and after the sperm selection process. **d** Top-down view of the microfluidic device during sperm selection. The sperm were stained using a vitality kit to visualize the migration of live sperm from semen containing dead and nonmotile sperm (left end) toward the center of the chip (right end). Insets I, ii, iii show cross-sections of the 3D printed microchannels at the designated points, while iV shows the magnetic microbead zone top down. **e** Schematic representation of the major selection mechanisms

The motility-based MSSP includes stages 1, 2, and 4, as shown in Fig. 1. The device was prefilled with Sperm Rinse buffer from the center by injecting 1.5 mL through the central outlet using a 3 mL BD plastic syringe. A strip of AS-110 acrylic medical grade adhesive tape was then used to seal the central outlet. Then, 0.85 mL of liquified semen was injected into the device using a 1 mL BD plastic syringe, and the chip was left undisturbed at 36 °C (on a hotplate) for 5, 10, 15, and 20 min for concentration, motility, and vitality validation. Later, 15 min was used for DNA fragmentation, apoptotic and cryopreservation experiments as the optimal time for selection. After incubation, the tape was removed from the outlet port, and 150 µL was collected from the central outlet. In addition to the side-by-side comparisons between SU and microfluidic methods, an additional set of 5 devices was run for 15 min at room temperature (RT) to assess the impact of incubation temperature on the velocity profiles of sperm collected. To test the performance of the device on oligozoospermic samples, diluted semen samples were loaded into the device in the same manner described.

#### Combined motility and apoptotic sperm selection

The second iteration of the MSSP contained the same geometry of channels with an additional reservoir for holding superparamagnetic microbeads situated at the end of the microchannels between the end of the channels and the collection zone (Fig. 1d). The microfluidic device was prepared by first injecting 1 mL of 180 µm iron paramagnetic microbeads (Thermo Fischer Scientific) coated with dextran and suspended in Annexin Binding Buffer (Thermo Fischer Scientific). This solution was injected using a 1 mL syringe tip through a dedicated inlet hole located above the sperm trapping zone (above insert iV in Fig. 1d). This inlet hole was then sealed with tape. The device was then prefilled with Annexin Binding Buffer in the same manner described above. The center outlet was covered with a strip of tape. A total of 0.455 mL of the liquified semen was mixed with 0.425 mL of MACS ART Annexin V reagent (Miltenyi Biotec), incubated at room temperature for 15 min, and injected into the device from the semen inlet located at the outer ring of the device. The device was then placed between two opposing neodymium magnetic plates (AMF Magnetics), which were positioned above and below the device to create a magnetic field and left for 15 min. Once the incubation finished, the tape was removed, and 150 μL of the selected sperm was immediately collected while keeping the device between the magnets.

### SU Method

The SU method used was appropriated from previous studies showing lower DNA fragmentation in SU than DGC^12,34^. After liquefaction, 1 mL of the semen sample was gently layered with 1.5 mL of pre-equilibrated Sperm Rinse media (Vitrolife) and placed in an incubator at 37 °C and 5% CO_2_. The tube was incubated for 45 min inclined at an angled position, which allowed the motile sperm to swim into the overlaid medium. After incubation, 0.9 ml of the upper layer was obtained and transferred to a clean tube where 3 mL of Sperm Rinse media was added and mixed. Then, the samples were washed by centrifugation at 500 × *g* for 7 min, the supernatant was discarded, and the pellet was resuspended in 100 μL of G-IVF Plus media. To test the performance of SU on oligozoospermic samples, diluted semen samples were layered in the same manner described.

### Sperm cryopreservation

Sperm cryopreservation was performed by first aliquoting freezing medium (Vitrolife) and leaving it to equilibrate to room temperature. Sperm to be frozen were then separated into different test tubes, one for each group (Raw, SU, MSSP, and H-MSSP). The sperm samples were diluted with freezing medium 1:1 (v/v) by adding the freezing media dropwise with 1 mL pipette. Following this, the mixture was left to equilibrate for 3 min and left at room temperature for 10 min. Mixtures were then transferred into 1 ml cryo-tubes suspended horizontally for 30 min, 5 cm above the surface of the liquid nitrogen (LN2). Finally, the cryo-tubes were plunged into the LN2 (−196 °C) with the cryotube upside down. The samples were cryopreserved for 7 days before thawing for reassessment. To thaw the sperm, the cryotube was placed in a 37 °C water bath for 1 min. Seven second videos were then recorded for motility analysis of the recovered sperm.

### Sperm DNA analysis

The DNA fragmentation index (DFI) was assessed by a modified sperm chromatin dispersion (SCD) test using the HT-HSG2 kit (Halotech DNA Pty Ltd) as previously reported^33^. The DFI of sperm was obtained before and after each sperm selection method. To perform the SCD assay, 90 μL of sperm suspension was added to an Eppendorf tube and mixed with prewarmed agarose. Ten microliters of the semen-agarose mixture was pipetted onto supercoated slides and covered with a coverslip. The slides were placed on a cold plate at 4 °C for 5 min to set the agarose. The coverslips were gently removed from the slides, and the slides were immediately immersed horizontally in an acid solution (from the kit) and incubated for 7 min. The slides were then gently tilted vertically to allow the acid solution to run off the slides. The slides were horizontally immersed in 10 mL of the lysing solution for 20 min and then washed with distilled water for 5 min. The slides were then dehydrated in increasing concentrations of ethanol (70%, 90%, and 100%) for 2 min each, air-dried, and stored at room temperature in the dark. To add color to the cells, slides were horizontally covered with a mixture of Wright’s staining solution (Merck) and phosphate-buffered saline (1:1, Merck) for 5 min and then briefly washed in DI water. Sperm were counted under brightfield microscopy using an Olympus Ix73 inverted microscope with an Olympus DP80 camera at ×20 magnification. A minimum of 300 spermatozoa per sample were scored. SCD analysis was performed by counting the number of sperm with and without visible halos as per the test manufacturer’s instructions. Sperm cells without a halo or with a weakly stained, small, or degraded halo were considered to have fragmented DNA, while sperm cells with medium to large halos were considered to have intact DNA. DFI is expressed as the percentage of sperm cells with fragmented DNA.

### Sperm concentration, vitality, and motility analyses

Sperm concentration and progressive motility were assessed manually after collection for each selection method according to the World Health Organization manual (fifth edition). The assessment of sperm vitality and additional motility characteristics were performed using previously reported methods^30,33^. Briefly, vitality was assessed using the fluorescence-based LIVE/DEAD sperm viability kit (Thermo Fisher Scientific) by staining live and dead sperm according to the supplier manual. A hemocytometer was used for counting and observed through an Olympus IX73 inverted microscope equipped with an Olympus DP80 camera for fluorescent imaging. Sperm motility parameters, including curvilinear velocity (VCL), straight line velocity (VSL), average path velocity (VAP), linearity (LIN), straightness (STR), beat cross frequency (BCF), and amplitude of lateral head displacement (ALH), were evaluated with the OpenCASA (Open Source Computer Aided Sperm Analysis) plugin in ImageJ (version 1.80) originally developed by Alque ´zar-Baeta et al.^35^. Analysis was performed using 5-s videos of sperm swimming in their collected media before and after each selection method (with a frame rate of 30 frames per second for each video). The CASA settings used were set to those from previously reported studies using the same CASA system^36^.

### Annexin V/PI binding assay and flow cytometry analysis

The technique used for the Annexin V assay was adapted from a previously reported method^21^. Spermatozoa were incubated in Annexin Binding Buffer (Biolegend) that contained FITC-labeled AAVV (0.1 mg/mL [w/v]) and PI (1 μg/mL [w/v]) (Sigma Aldrich). A negative control sample was suspended in HEPES A buffer only. After exactly 15 min at −20 °C, the spermatozoa were analyzed in a CytoFLEX LX flow cytometer system and CytExpert software. A minimum of 8000 spermatozoa were examined for each test. The sperm population was gated by using forward-angle light scatter; side-angle light scatter was used to exclude electronic noise and debris. All tests were run in triplicate.

### Statistical analysis

All statistical analyses were performed using GraphPad Prism 6.0 (GraphPad Software). The statistical significance of the differences between the values was assessed using the Friedmans test for nonparametric data. *P* < 0.05 was considered statistically significant.

## Results

### Device geometry and operation

Figure 1 illustrates the workflow of our device, in which sperm can migrate from a semen reservoir into microchannels preloaded with sperm buffer. Each microchannel contains layers of grooves and ridges to help double the number of avenues and corners for sperm to follow when compared to that of conventional straight channels^30,37^. The channels also provide a larger entry space compared to that of previous iterations of the device, so more sperm find their way into the channels for selection^30,37^. The geometry of the channels was optimized first by device stability and then by motile sperm yield. The width of the grooves and the height of each channel were incrementally increased to a point where the device consistently yielded over 60% more sperm than that of the original geometry^30^ without allowing semen to penetrate the channels during the semen injection step. Furthermore, we also present a second iteration of this microfluidic platform in which sperm, after being premixed with Annexin-coated magnetic nanoparticles, are sorted based on motility before being introduced to a magnetic field amplified by paramagnetic microparticles, effectively trapping phosphatidylserine-positive sperm. Finally, sperm are collected from the center outlet.

### Sperm DFI and conventional quality metrics

**Fig. 2:**
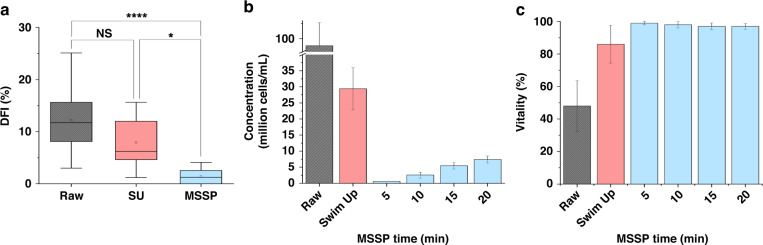
Sperm quality metrics comparing neat semen, SU, and microfluidic sperm selection methods. **a** DFI of side-by-side testing between SU and microfluidic selection methods where the microfluidic device was left to incubate for 15 min (*N* = 19). **b** Concentration of sperm collected from the device across different time points compared to the SU method and raw semen. The bar graph is split to better illustrate the concentration of sperm from the device. The samples were identical to those used for DFI and those used for vitality staining (*N* = 24). **c** Vitality of sperm from LIVE/DEAD staining between raw, SU and microfluidic sperm populations (*N* = 5). Error bars represent the standard deviation within the sample

Sperm DFI, concentration, vitality, and motility values were compared before and after SU and microfluidic sperm selection methods. Samples greater than 2.1 mL were split without dilution between SU and microfluidic selection methods, while samples between 1.1 and 2.1 mL were split to produce 0.1 mL of neat semen for raw sample analysis, and the remaining volume was split 50/50 by volume and diluted up to 1 mL before selection. Samples less than 1.1 mL were not included in the study. Among the 33 volunteers who participated, 25 had semen volumes equal to or greater than 2.1 mL, with 5 of the donated semen classified as infertile according to the WHO 5^th^ Edition criteria. The average DNA fragmentation of all raw samples was 12.19% (±5.59) and ranged from 3.0% to 25.12% (Fig. 2). The results show that DFI values of semen samples prepared through the microfluidic method (1.44% ± 1.4) were significantly lower than those prepared using the SU method (7.92% ± 4.72, *P* = 0.0176). This represents an average DFI reduction of 88.2% and 35.0% for MSSP- and SU-selected sperm, respectively.

For each experiment, 150 µL of isolated sperm was recovered from the microfluidic device. The microfluidic method provides a significant time improvement from conventional SU-based techniques. Sperm concentrations from the device increased with incubation time and ranged from an average of 0.61 million sperm/mL after 5 min of semen incubation to 1.54, 5.46, and 7.4 million sperm/mL after 10, 15, and 20 min, respectively. For 15 min of microfluidic device incubation, the average number of sperm collected from the device was 825,000 sperm, sufficient for droplet-based IVF and more than enough to select an individual sperm for intracytoplasmic sperm injection (ICSI). In addition, the vitality of sperm from the devices was assessed to confirm that no adverse effects on the viability of cells occurred from the materials used in the device. Sperm vitality remained above 97% for all incubation times and was consistently greater than that of the raw semen (48.1% ±15.6) and SU (86% ±11.6) method.

**Fig. 3:**
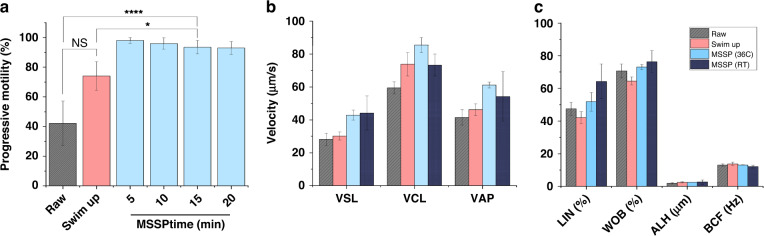
Motility parameters between raw semen, SU, and microfluidic separation methods. **a** Progressive motility of sperm processed by SU and microfluidic methods compared to neat semen samples. Microfluidic tests were split into 5-, 10-, 15-, and 20-min selections. **b** Velocity parameters of sperm from the same aforementioned groups plus devices operated at room temperature, as assessed by OpenCASA. **c** LIN, WOB, ALH, and BCF of sperm from the same aforementioned groups. (*N* = 19 for Raw, SU and microfluidic (36 C) and *N* = 5 for microfluidic (RT)). The devices were incubated for 15 min each in both (**b**) and (**c**). Error bars represent the standard deviation within the sample

Sperm motility was also assessed for each method and compared to the raw semen. This was performed across four different incubation times for the device (5, 10, 15, and 20 min). The average progressive motility from the microfluidic device (93.5% at 15 min) exhibited a statistically significant increase compared to that of raw semen (37%, *P* = 0.001) and the SU method (74.1%, *P* = 0.0181) (Fig. 3a). Considering the high yield, vitality, and motility of sperm from the microfluidic device at 15 min, 15 min was chosen as the ideal time for DFI-based experiments, cryopreservation experiments, and apoptotic selection-based experiments. The OpenCASA plugin in ImageJ was used to quantify sperm motility parameters. Straight line velocity (VSL) is the averaged velocity of a sperm over time along the straight line between its first and last screen positions. Curvilinear velocity (VCL) is the average velocity of a sperm tracked along the actual point-to-point path the cell took. The average path velocity (VAP) measures the sperm along the smoothed VCL trajectory. Linearity (LIN) is defined as the linearity of a sperm trajectory calculated by the equation VCL = VSL/VCL × 100. The amplitude of lateral head displacement (ALH) is the maximum lateral displacement of a sperm head as it moves along its average trajectory and provides an assessment of track width. Beat cross frequency (BCF) is the averaged rate at which the sperm curvilinear track moves over its averaged path trajectory^38^. The straight, curvilinear, and average path velocities for sperm separated via microfluidics at 36 °C (44.2 ± 10.4, 73.3 ± 6.7, and 54.2 ± 15.2 µm/s) each showed a considerable average increase from that of the raw semen (28.17 ± 3.78, 59.47 ± 3.68, and 41.48 ± 4.76 µm/s) and from the SU method (30.2 ± 2.5, 73.8 ± 7.1, and 46.2 ± 3.6 µm/s) (Fig. 3b). Both room temperature (22 °C) and body temperature were compared for microfluidic sperm selection (at 15 min incubation) to compare the differences in sperm motility behavior upon collection, which may affect their level of hyperactivity, capacitation, and suitability for conventional IVF. The room temperature microfluidic sperm separation showed a similar increase in VSL (as the device incubated at 36 °C) but was comparable to the SU method in terms of curvilinear velocity and showed a higher range of average path velocity values. Similarly, the LIN and WOB showed average increases with microfluidic sperm selection compared to that of the SU method and the neat semen (Fig. 3c). However, ALH and BCF were largely unchanged between all groups. Interestingly, the SU method demonstrated a loss in LIN, WOB, and STR from the neat semen, while the RT microfluidic separation produced the largest increases, which exceeded that of microfluidic separation incubated at body temperature.

In addition to processing raw semen samples, to characterize the performance of SU and the device for oligozoospermic samples, each method was tested with diluted samples with a sperm concentration below the reference limit of 15 million sperm per mL outlined by the WHO 6th edition guideline^1^. All results for oligozoospermic samples can be found in Supplementary Fig. 3 and Supplementary Table 3. Sperm concentrations from the device (with a 15-min incubation) averaged just above 1 million sperm/mL compared to the 1.8 million sperm/mL from SU (Supplementary Fig. 3A). The average progressive motility from the microfluidic device was 96.5%, which was still a significant increase compared to that of raw semen (39.9%, *P* = 0.001) and the SU method (85.1%, *P* = 0.021) (Supplementary Fig. 3B). DNA fragmentation also followed a similar trend to nonoligozoospermic samples, in which the device yielded an average 1.3% DNA fragmentation (±1.1%), the SU yielded a significantly higher value of 6.8% (±3.8, *P* = 0.018), and the raw average was 11.2% (±4.9, *P* < 0.0001) (Supplementary Fig. 3C). Sperm vitality remained high at 99% for the device, which was an improvement over the raw semen (44.5% ±16.4) and SU method (89.1% ±9.0) (Supplementary Fig. 3D).

### Characterization of apoptotic sperm and negative selection of apoptotic sperm from the hybrid MSSP (H-MSSP)

**Fig. 4:**
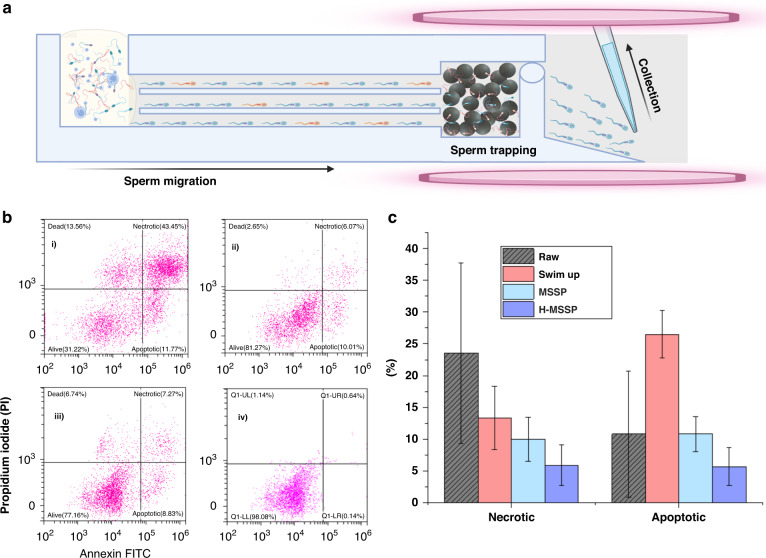
Overview of the mechanism and output from H-MMSP sperm selection when compared to raw, SU, and MSSP sperm. **a** Schematic representation of the H-MSSP workflow through magnetic immobilization of apoptotic sperm. Sperm move from the raw semen in the buffer-filled channels and are guided by ridges toward the trapping zone, where apoptotic sperm are trapped through the magnetic force of magnets above and below the device. Nonapoptotic sperm can proceed for collection. **b** Representative flow cytometry images from each group of stained (PI and AAV-FITC) sperm from the aforementioned groups showing the effective reduction of apoptotic sperm from hybrid MSSP. (I) Raw semen, (ii) MSSP, (iii) SU, (iV) H-MSSP (*N* = 5). **c** Apoptotic and necrotic sperm percentages from raw, SU and microfluidics-selected sperm

To investigate the incidence rate of sperm apoptosis caused by each selection method and to reduce the number of apoptotic sperm at collection, sperm apoptosis and DFI were measured for raw, SU, motility only and hybrid MSSPs. For the removal of apoptotic sperm from collected sperm samples, a magnetic sperm selection approach was used to trap prelabeled apoptotic sperm using a magnetic field amplified by superparamagnetic microbeads (Fig. 4a). Raw, SU, MSSP only, and hybrid MSSP with magnetic separation were compared between 5 samples of larger volume (>2.5 mL). The percentage of apoptosis was assessed via flow cytometry using PI and AAV-FITC double staining. The percentage of apoptotic sperm (Fig. 4c) showed a large average increase in SU-sorted sperm when compared to that of the raw sperm (8.5% to 26.5%). While motile sperm selection via the MSSP only group showed no average reduction in apoptosis, the hybrid MSSP showed a near 50% reduction in apoptotic sperm versus the MSSP only sperm. Necrotic or late apoptotic sperm were reduced in all sperm selection methods (Table 1). Figure 4b shows an example distribution for the relative decrease in alive (AAV−/PI−), dead (AAV−/PI+), necrotic (AAV+/PI+) and apoptotic (AAV+/PI−) sperm populations between each method with the raw sample (with between 1% and 25% total apoptotic sperm). DFI remained low in the hybrid MSSP at 0.7%, which was comparable to the regular MSSP.

**Table 1:** Sperm assessments before and after SU and microfluidic sperm selection at 15 min

Sperm metric	Raw semen	Swim-up	MSSP	Hybrid MSSP
Concentration (×10^6^)	87.7 (±39.2)	29.4 (±6.6)	5.5 (±0.15)	7.83 (±0.23)
Total motility (%)	45.4 (±14.3)	82.2 (±10.2)	95.5 (±3.1)	97.6 (±2.5)
Progressive motility (×10^6^)	42.2 (±15.0)	74.1 (±9.6)	93.5 (±4.5)	97.0 (±2.9)
Sperm vitality (%)	48.1 (±15.6)	86.0 (±11.6)	97 (±2.1)	98.5 (±1.1)
DNA fragmentation (%)	12.2 (±5.6)	7.9 (±4.7)	1.4 (±1.4)	0.7 (±1.5)
Apoptotic sperm (%)	10.8 (±9.9)	26.5 (±2.7)	10.8 (±2.8)	5.66 (±3.0)
Necrotic/late apoptotic sperm	23.6 (±14.2)	13.31(±1.5)	9.94 (±3.5)	5.85 (±3.2)
Cryosurvival (% motile)	39.8 (±8.1)	55.7 (±12.2)	67.5 (±12.5)	73.2 (±11.0)
Cryosurvival (Vitality %)	59.9 (±7.5)	53.8 (±6.0)	66.8 (±7.7)	71.1 (±9.3)

### Motile sperm recovery following sperm cryopreservation

**Fig. 5:**
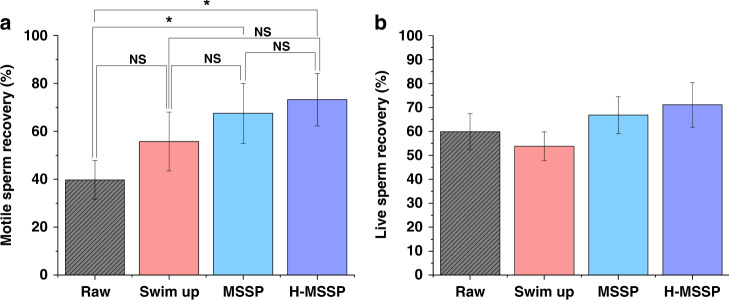
Motility characteristics of sperm before and after cryopreservation from the raw, SU and microfluidic groups (N = 5). **a** Percentage of motile sperm recovery following sperm cryopreservation. **b** Recovery percentages of live sperm via LIVE/DEAD following sperm cryopreservation. Error bars represent the standard deviation within the sample

To investigate the effect of sorting on motile sperm recovery following sperm cryopreservation and the influence of different separation methods on sperm recovery, sperm cryopreservation was performed on raw semen, SU, MSSP, and H-MSSP sorted sperm. Sperm selections were performed side-by-side and subsequently cryopreserved for 7 days before being thawed alongside an aliquot of raw semen. As shown in Fig. 5a, sperm processed through MSSP (on average) showed a significant 70.0% improvement (*P* = 0.0324) in motile sperm recovery when compared to that of the raw conventionally frozen sperm (39.7–67.5%). Sperm processed through the hybrid MSSP platform also showed a significant 84% improvement (*P* = 0.0286), with an average 73.2% motile sperm recovery. SU processed sperm showed a nonsignificant average improvement of 40.1% (39.7–55.8%). On the other hand, no significant difference was observed for the recovery of live sperm post-thawing between each group, with a higher average value for both microfluidic variants (Fig. 5b). Sperm frozen from a raw semen dilution showed an average recovery of 59.9%, while the SU, MSSP, and hybrid MSSP-processed sperm exhibited average recoveries of 53.8%, 66.8%, and 71.1%, respectively. OpenCASA revealed differences in the post-thaw velocity parameter of sperm from different groups (Supplementary Fig. 2A, B). SU processed sperm showed the largest and most consistent decrease in velocity across VSL, VCL, and VAP, while unprocessed and MSSP sperm showed very minor changes in velocity in all parameters except VAP, in which the raw sperm showed an increase.

## Discussion

### Sperm quality metrics before and after selection

We have developed a 3D printed microfluidic sperm selection platform (dubbed the MSSP) for the simple selection of high-quality sperm, and this platform exhibits significantly improved DNA integrity, motility, vitality, and cryo-survivability compared to that of a conventional SU-based approach. Comparative studies to conventional gold-standard methods are key to translating new techniques and until now remained uninvestigated in previous iterations of the MSSP^30,37^. SU was chosen for this study because it is the most comparable to microfluidic motility-based selection and because studies report a more effective reduction in sperm DNA fragmentation compared to that of DGC^34,39–41^. However, due to the global popularity of DGC and its functionally different selection mechanism, further comparative testing against DGC, SU, and DGC plus SU combinations will be necessary for clinical adoption. Microfluidics has been increasingly involved in ART studies, and several devices have been explored to sort motile or morphologically intact sperm from unprocessed semen^33,42–47^. However, the translation of these technologies into clinics has been extremely limited, largely due to their complexity in operation, instability, and/or inconsistency, which often result from mechanisms such as gravity pumps, laminar flow, or charge-based selection. Without an intuitive user interface, many devices have not been tested side-by-side to evaluate their performance. A successful sperm selection platform must provide high-quality sperm in a timely manner, be simple to use and exhibit consistent performances. The platform we report here is a passive, easy-to-use platform that selects sperm based on their tendency to follow guidelines and turn corners; at the same time, sperm are resuspended in sperm nutrient buffer without the need for centrifugation.

This newer geometry builds upon previous studies^30,37,48^ by using 3D printing to increase the number of boundaries (up to 3-fold) and size of channel openings for sperm to be guided out of seminal plasma and into fresh media for collection. The walls and grooves of the device are inspired by the folds, grooves, and tight openings that sperm must traverse in the cervix and the uterotubal junction^5,49^. Mimicking these structures resulted in a 68.4% improvement in total sperm count (490,000–825,000) from the device for the same incubation time (15 min) compared to that of the previously reported geometry by Nosrati et al. Notably, this was achieved using a lower average starting concentration from raw semen (87.7 million/mL compared to 120 million/mL). Compared to nonoligozoospermic samples, both the device and SU suffered a reduction in sperm yield, with the SU exhibiting a 9.5% reduction in yield (33.5–24.4%) and the MSSP showing a 6.2% increase in yield (6.3–12.5%). This indicates that the device still captured and retained sperm from oligozoospermic samples; however, further testing on more clinically oligozoospermic samples is needed. 3D printing enabled the rapid iteration of complex geometries to maintain the stability of the device during sample loading and improve sperm yield. This was aided by the fluid boundary present at the entry to the device’s channels (visible in Fig. 1d where the raw semen in orange meets the entrance of the microchannels), at which sperm move from the viscous semen into the sperm buffer before selection; this movement is similar to how sperm exit the seminal plasma (deposited in the vagina) and quickly migrate into the female fluids^5,49^. However, although the 3D printed devices showed no adverse effects on sperm vitality, DNA fragmentation, or apoptosis, 3D printing is not a scalable manufacturing method. For the commercial application of this device, a layered microinjection molding approach in either COC or PMMA (both currently FDA-, CE-, and TGA-certified materials for sperm selection) would be needed. Although the MSSP does not reach the number of sperm isolated from the SU method, the results were significantly improved from previously reported results^30,37^, bringing sperm concentrations to a clinically useful level. A strong correlation between high DNA integrity and the tendency for sperm to follow boundaries has been previously reported by Nosrati et al.^37,50^. In these studies, when sperm followed boundaries, a near 80% improvement in DNA integrity was observed compared to the starting value in raw samples only^30^. However, to date, side-by-side studies comparing microfluidic sperm selection against the conventional motility-based selection method, SU, are few and far between. Furthermore, the effects of cryopreservation on selected sperm via microfluidics have not been frequently investigated. Previous studies have claimed that neither SU nor DGC are suitable methods for preparing high DFI samples, as they often cannot reduce the DFI to an acceptable range (<15%) or, in some cases, may increase the incidence of DNA damage through ROS generation and iatrogenic damage^12–14^. In this study, microfluidic sperm sorting consistently resulted in a significant reduction in sperm DFI in all samples and, in several cases, reduced the DFI by 100%. The average DFI reduction from MSSP sorted samples was 88.2% and 35.0% for SU-selected sperm, which is consistent with previously reported studies on conventional SU-based approaches^29,34,42^. While SU did not increase the DFI in any samples, it did show a wide variance of improvements, which contrasts with the narrower range of DFI results from the MSSP, regardless of the DFI starting value. The highest raw DFI tested was 25.12%, in which the MSSP was reduced to less than 2%. While further testing with DFI values greater than 30% is still needed, the low standard deviation of DFI values from MSSP sorted sperm indicates that (unlike SU) the initial level of DNA fragmentation is irrelevant. DFI values play an important role during blastocyst development and have been shown to play a prominent role in embryo implantation and miscarriage rates^6,51,52^. To reduce the risk of miscarriage after ICSI or IVF, a reliable and simple method to select sperm is needed. This study shows that MSSP is effective for selecting sperm and causes little to no DNA damage in semen samples with motile sperm populations.

With many microfluidic methods, the yield of sperm is often only suitable for ICSI, and many devices collectbetween 3000 and 400,000 sperm per selection^44,53^. The microfluidic device in this study was designed with a three-dimensional geometry to encourage multiple layers of sperm boundary-following behavior and increase the number of sperm in the semen that interact with the microchannels without compromising the stability of the chip. This increases sperm throughput by mimicking the grooves and fold sperm traverse in vivo, providing an improvement over the conventional straight channels used in previous studies^30,48,54^. The sperm are also contained safely within the device rather than in an open dish, such as seen in the FertDish^48^, which limits spill risk and provides a robust method of collection that requires less operational skill. The fabrication method used in this study relied upon 3D printing to create cantilever ridges. Thus, total sperm counts of approximately 825,000 sperm were obtained when the device was incubated for 15 min, which is tunable to the quality of the unprocessed semen sample. Lower quality semen samples with lower motile sperm concentrations may require longer incubation times to achieve similar sperm yields, or technicians may opt for shorter times for ICSI patients in which only a small population of high DNA integrity sperm are needed. Considering that the standard ratio for IVF in humans is 50,000 or more motile sperm per oocyte^55^, the average yield of sperm from MSSP represents a clinically useful platform for both IVF and ICSI. However, it is worth noting that the lower limits of the sperm-oocyte ratio for achieving fertilization heavily depend on sperm quality. Therefore, considering the high level of DFI reduction from microfluidic sperm selection, further investigation into the appropriate sperm-oocyte ratio would be prudent. Furthermore, this study recovered semen samples from a limited number of infertile donors, and a study dedicated to the processing of low motile sperm populations is needed.

To ensure that there were no adverse toxic effects on sperm vitality, staining was performed and showed no detrimental effects on sperm vitality after selection. However, SU yielded an average of 14% PI-stained sperm with a high variability compared to that of MSSP (3%), which indicates a less standardized process prone to human error when compared with DFI and motility. Compared to SU, microfluidic-based sperm selection involves less manual interventions and provides a threefold reduction in sperm incubation time (45 min down to 15 min).

Sperm motility is an important metric for assessing the quality of sperm and their ability to fertilize an oocyte, particularly in IVF. Motility after SU typically lies within the range of 65–85%, which is consistent with the average motility for SU-prepared sperm in this study^34,42,56^. Although SU provided a general improvement in progressive sperm motility, it was significantly outperformed by the microfluidic sperm selection variations regardless of the incubation time for the device. Furthermore, the grade of sperm motility may vary in terms of velocity profile (VCL, VSL, and VAP) and other motility characteristics (LIN, WOB, ALH, and BCF). All velocity parameters showed greater improvement in microfluidics-selected sperm than that of SU sperm, demonstrating a much higher proportion of grade A sperm, particularly when the device was incubated at 36 °C during selection. This is due to the boundary-following behavior mechanism leveraged within the device, which promotes the migration of highly motile sperm. Sperm selected at RT for the MSSP showed similar improvement in VSL but less so for VCL and VAP, which combined with a large level of linearity. Figure 3c shows the linearity of microfluidics-selected sperm increase, which again indicates that sperm from the microfluidic methods produce more energy and potentially more fecund, as these sperm (in vivo) are more likely to penetrate the cervical mucus, traverse the uterotubal junction, and result in increased fertilization rates^57^. However, none of the variations are indicative of sperm hyperactivation or capacitation, in which sperm swim at speeds two- to threefold faster and experience an increase in ALH and head oscillations.

### Hybrid sperm selection

Recent reviews and studies have recognized the importance of moving beyond solely motility-based sperm selection methods toward combinational approaches that use either negative or positive sperm selection based on the expression of certain biomarkers^5,19,58^. Conventional methods of selection (SU and DCG) omit the selection of sperm that express any kind of biological moiety, show abnormal sperm function or a lack of fecundity that may impact downstream processes in fertility treatment, such as embryo quality or live birth rate. Existing microfluidic methods also typically rely on a single mechanism for sperm selection and do not possess the (optional) capability to provide more rigorous selection criteria within a single platform without dramatically increasing selection time or complexity. One such marker is the externalized membrane protein phosphatidylserine, which is an indicator of early-stage apoptosis in sperm. It has already been proven that the proportion of early apoptotic sperm cells in raw semen can reach over 20%^21^ and that eliminating apoptotic sperm using annexins correlates to an improvement in embryo quality^22^ and a further decrease in sperm DNA fragmentation, which is directly correlated to apoptosis^59^. High levels of early-stage sperm apoptosis have also been suggested to play a role in recurrent pregnancy loss^25^. It has also been demonstrated that utilizing apoptotic sperm removal plays a significant role in improving the fertilization rate and rates of clinical pregnancies when combined with the use of good-quality donor oocytes^22^. Removing apoptotic sperm is valuable because they could still fertilize an oocyte, particularly during an ICSI treatment, as they often present without morphological abnormalities. However, neither DGC nor SU methods can effectively remove apoptotic sperm populations, and the only clinically viable method to achieve this is the application of magnetic-activated cell sorting (MACS)^29^. However, the MACS process on its own compounds the time and cost of clinical sperm selection, bringing the total process to well over 2 h per sample, which is not practical in a clinical setting.

To remedy this issue and provide a biologically inspired approach to sperm selection, we designed a microfluidic platform for dual-action sperm selection, in which the optional inclusion of apoptotic sperm removal is possible when clinically relevant. Our device first leverages sperm motility and boundary-following behavior, which is naturally observed in the cervix and uterotubal junction; then, sperm that express apoptotic markers are trapped, which is thought to naturally occur in the male reproductive tract during spermatogenesis^5^. While motility-only MSSP did not increase the incidence rate of apoptosis in sperm post selection, SU-sorted sperm showed an average 3-fold increase in sperm apoptosis. This indicates that the SU method induces apoptosis in sperm, which can be seen by the cluster of cells in the lower right quadrant of the flow cytometry data (Fig. 5c). In comparison, the hybrid MSSP, which retains apoptotic sperm in the device, showed a marked reduction in AAV-positive sperm, as indicated by the reduction in cells present in the lower right gated quadrant. It also showed a similar reduction in possibly necrotic or late apoptotic sperm (AAV+, PI+) from the motility-only microfluidic method. Raw semen contained sperm of all categories, which was expected. However, testing samples from infertile patients with more abnormal semen parameters may reveal an even higher abundance of apoptotic or late apoptotic expression. Several studies have indicated that removing apoptotic cells through MACS provides a beneficial effect on the pregnancy rate when compared to DGC and SU^29^. Although it remains unclear whether these benefits extend to the implantation rate or miscarriage rate, it is concerning that conventional methods such as SU appear to increase rates of sperm apoptosis (Fig. 5). Microfluidic hybrid sperm selection as opposed to addons such as MACS is beneficial because no additional operation or sperm incubation time is needed, potentially reducing cost and the chance for human-induced operator error. This also represents a departure from conventional microfluidic solutions, which typically employ a single selection mechanism, such as unguided motility^46^ or motility-based guidance alone^48,54,60^. Based on this, despite the limited number of samples tested, the use of biomarkers for sperm selection warrants further investigation and a larger number of experiments. The AAV-phosphatidylserine interaction is just one potential interaction that could be leveraged within this platform and may be appropriated to include more discerning sperm biomarkers in the future.

### Sperm cryopreservation

Another important component of ART cycles is the use of cryopreserved semen and their post-thaw characteristics, particularly motility. Despite the well-documented effects of sperm cryoinjury resulting from sperm cryopreservation, the cryopreservation of human sperm is becoming more commonplace, particularly for those undergoing treatment for cancers or for sperm donation due to the rising number of same sex and single parent cycles. During cryopreservation and thawing, sperm are subject to osmotic and oxidative stress as well as the formation of ice crystals, resulting in losses in motility and vitality typically between 40% and 50%^61,62^. Limited studies have reported the benefits of performing sperm sorting before cryopreservation^63,64^. However, here, the cryopreservation and subsequent thawing of sperm from both microfluidic platforms showed a marked improvement in recovered motility. Although the post-thaw recovery of living sperm from the MSSP was similar to that of sperm from diluted raw semen samples, a significantly higher percentage of motile sperm was recovered. Interestingly, the H-MSSP showed a greater improvement in post-thaw motility and vitality, which is consistent with other works, in which the removal or apoptotic sperm was demonstrated to improve cryo-survivability^65–67^. SU processed sperm showed an average reduction in sperm vitality but a nonsignificant increase in average motile sperm recovery. The velocity parameters of MSSP-processed sperm showed a greater variance in VSL and VCL compared to that of pre-freezing but retained the improvement in velocity when compared to that of SU, which decreased post-thaw. This indicates that sperm populations from microfluidic selection, which possess a higher percentage of grade A sperm, are more resistant to cryoinjury, particularly those with reduced levels of apoptotic expression.

### Future directions

The microfluidic selection platforms presented in this study provide highly motile sperm with substantially reduced DNA fragmentation and improved cryo-survivability. The device also has the (optional) capability to perform biomarker-based filtering of sperm with undesirable characteristics, such as the expression of phosphatidylserine, which is an apoptotic marker. Both of these methods have been shown to outperform the SU technique in human semen samples but require further testing for clinical translation. Additional side-by-side testing against DGC and combinations of DGC + SU in clinically infertile sample populations with high DFI and low motile sperm concentrations will be critical to successful implementation. Furthermore, the use of scalable manufacturing practices in gamete biocompatible materials, such as layered microinjection molding of COC, will be necessary to produce this device in the volumes required for impactful commercialization.

## Conclusion

We have developed a simple biomimetic microfluidic sperm selection platform with the includable option for apoptotic sperm cell removal in a hybrid system. The device uses 3D printed ridges to select the boundary following sperm with low DNA fragmentation and high progressive motility and better motile sperm recovery post-cryopreservation. The MSSP device can efficiently and consistently prepare sperm, resulting in significantly lower DNA fragmentation and higher-grade motility than that of the SU method. The device performs sperm washing and selection simultaneously while also significantly reducing the number of apoptotic sperm in the collected sample, providing clinically relevant sperm concentrations for IVF or ICSI within 15 min. By reducing the number of manual operations and time down to one-third of conventional sperm sorting methods without using damaging centrifugal forces that risk iatrogenic injury to sperm, this platform shows potential as a technologically disruptive medical device for use in fertility treatments. Further research on the clinical use of the MSSP is needed to validate its usefulness in abnormal semen samples.

## Supplementary information

ESI

Sperm Migration
