# Supplementary material for: Sperm quality metrics were improved by a biomimetic microfluidic selection platform compared to swim-up methods
Source: Microsyst Nanoeng. 2023 Mar 28;9:37. doi: 10.1038/s41378-023-00501-7 (PMC10050147; doi:10.1038/s41378-023-00501-7)
Supplement: Supplementary file 1 — ESI [file 41378_2023_501_MOESM1_ESM.docx]

Electronic supplementary information

**A Biomimetic Microfluidic Selection Platform Providing Improved Sperm Quality Metrics Compared to Swim-Up** Steven A. Vasilescu^1^, Lin Ding^1^, Farin Yazdan Parast^2^, Reza Nosrati^2^, Majid Ebrahimi Warkiani^1,3*^

Herein is a detailed description of aforementioned methods and details from the main text. This supplementary file includes:

- Image detailing the differences in workflow between swim up and microfluidic-based sperm selection methods.
- Image showing cross-sectional images of the device along different point of the device above a fluorescent top view of the device in action using live/dead sperm staining after 15 minutes incubation
- Graphs detailing the motility characteristics of sperm from different selection methods via Open CASA
- Graphs detailing the sperm quality metrics of sperm from oligozoospermic samples
- Supplementary video 1 description


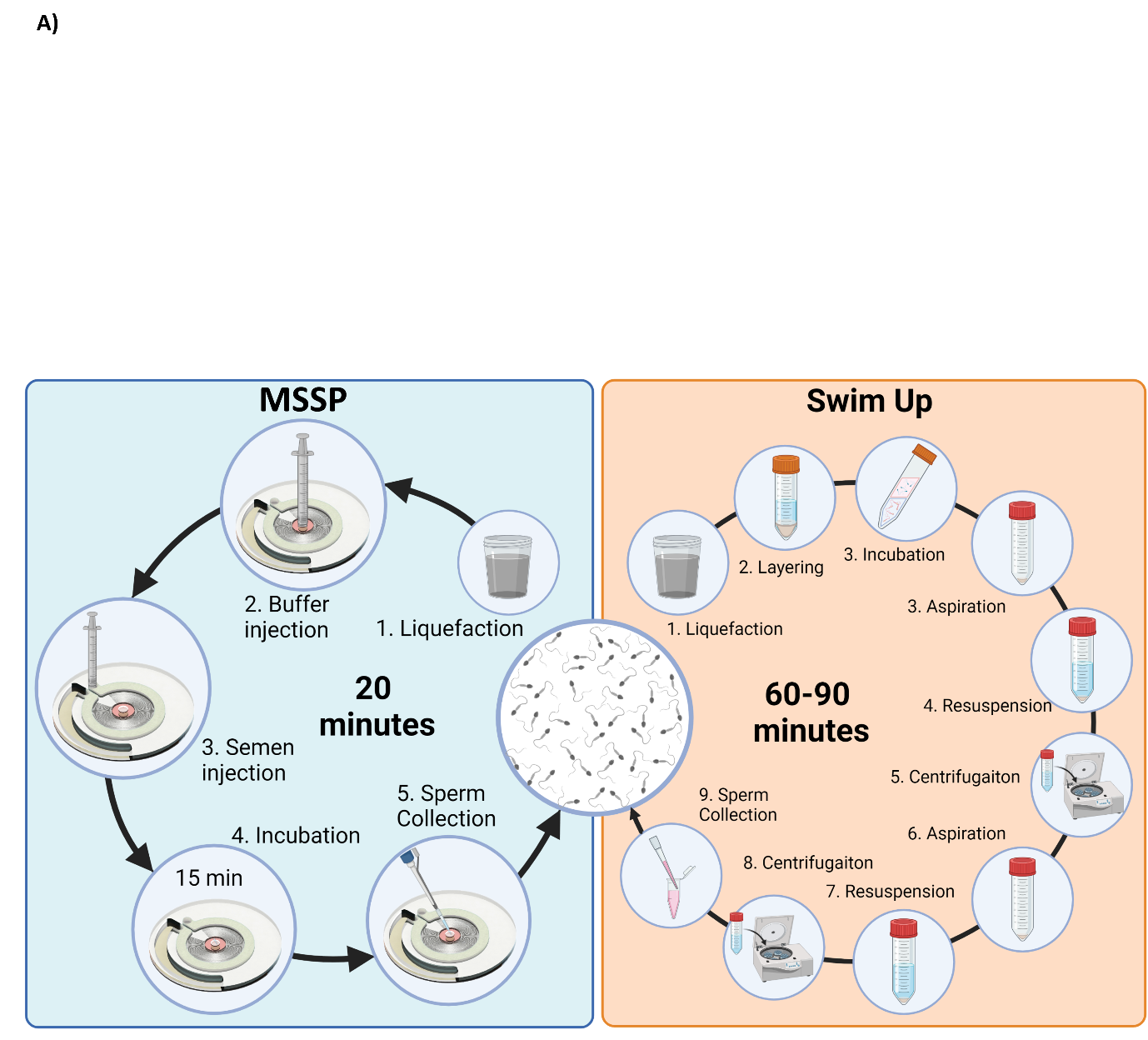


Figure 1. Schematic comparison of MSSP vs SU-based sperm selection methods by steps and time involved.


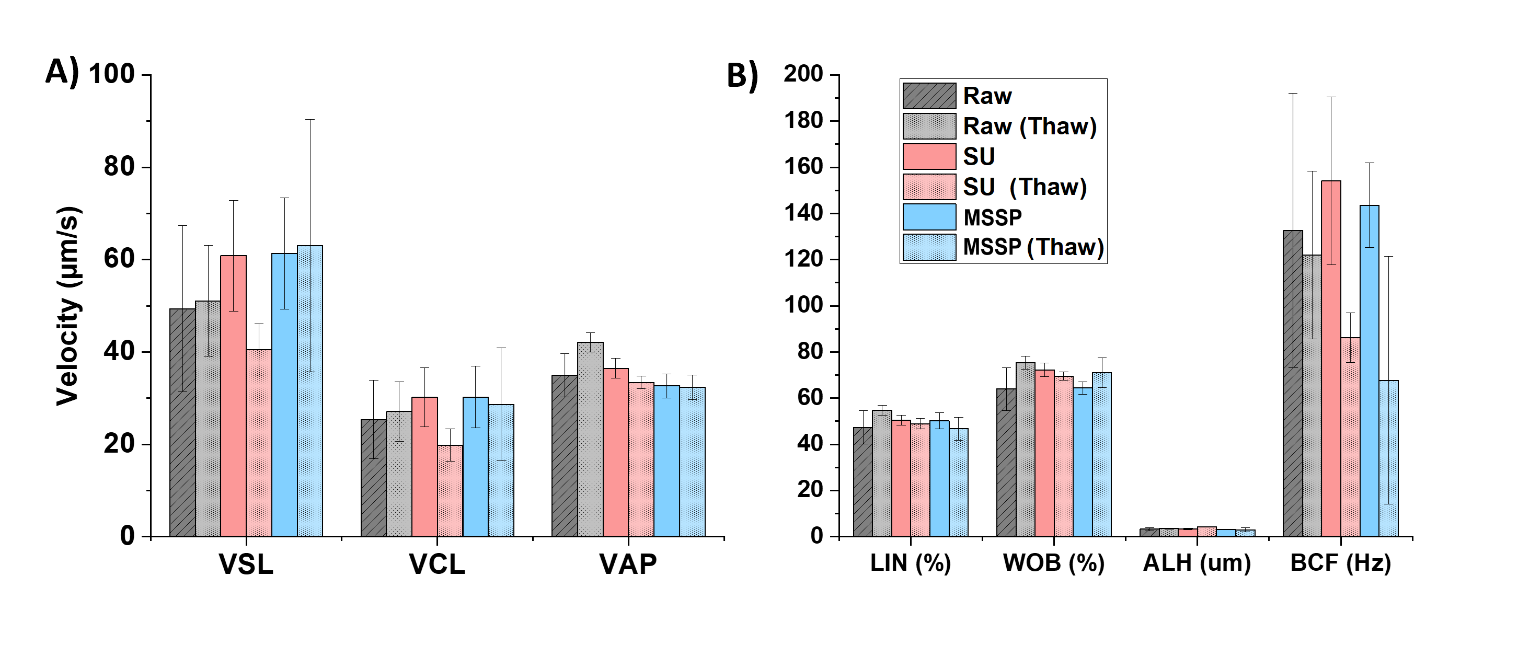


Figure 2. (A) Velocity parameters of sperm from Raw, SU and MSSP processed sperm before and after cryopreservation. (B) LIN, WOB, ALH, and BCF of sperm from the same aforementioned groups


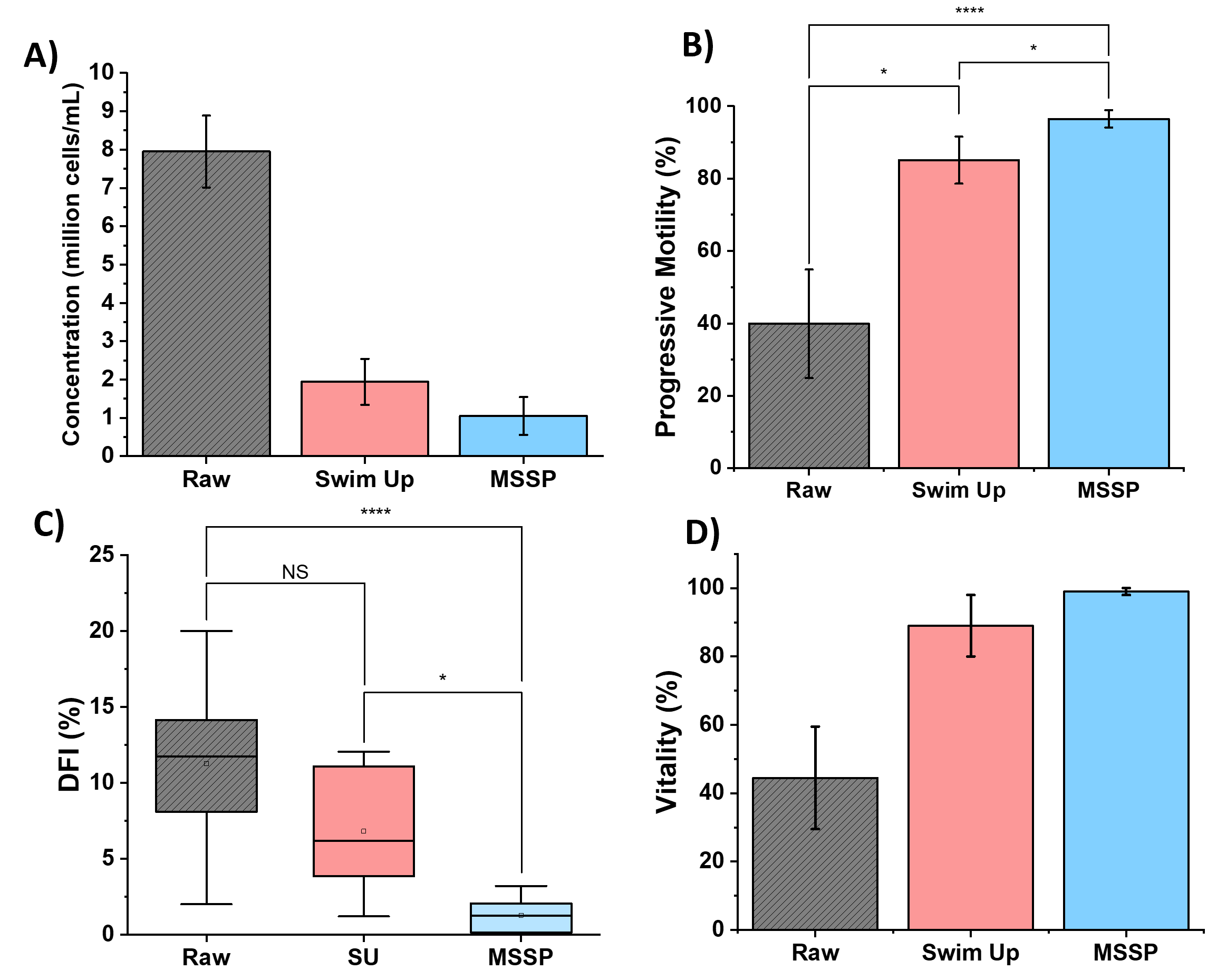


Figure 3. Sperm quality metrics comparing oligozoospermic (diluted) neat semen, SU, and microfluidic sperm selection methods. (A) Concentration of sperm collected from each method compared with diluted semen. (B) Motility parameters between raw semen, SU, and microfluidic separation methods (C) DFI of side-by-side testing between SU and microfluidic selection methods. (D) Vitality of sperm from LIVE/DEAD staining between raw, SU and microfluidic sperm populations (N=6). Error bars represent standard deviation within the sample. In each microfluidic sperm selection, the microfluidic device was left to incubate for 15 minutes.

Table 1 Motility parameters from OpenCASA for side-by-side sperm selection comparing SU and MSSP (at RT and 36C)

|  | **Raw** | **SD** | **Swim Up** | **SD** | **MSSP (36C)** | **SD** | | **MSSP (RT)** | **SD** |
| --- | --- | --- | --- | --- | --- | --- | --- | --- | --- |
| **VSL** | 28.17 | 3.78 | 30.178 | 2.47241 | 42.89233 | | 3.08697 | 44.1554 | 10.4028 |
| **VCL** | 59.47 | 3.68 | 73.7886 | 7.13269 | 85.37167 | | 4.64605 | 73.2808 | 6.67 |
| **VAP** | 41.48 | 4.67 | 46.1806 | 3.6065 | 61.16533 | | 1.75904 | 54.1552 | 15.17356 |
| **LIN (%)** | 47.55 | 3.9 | 42.1162 | 3.70987 | 51.87733 | | 5.67975 | 64.295 | 10.57751 |
| **WOB (%)** | 70.7 | 4.24 | 64.5406 | 2.43831 | 73.07833 | | 1.5799 | 76.3498 | 6.75359 |
| **STR (%)** | 66.56 | 4.06 | 64.4898 | 5.1623 | 70.34267 | | 6.15784 | 83.0362 | 6.74328 |
| **ALH (um)** | 1.9 | 0.43 | 2.4998 | 0.40618 | 2.477 | | 0.01512 | 2.6746 | 1.34375 |
| **BCF (Hz)** | 13.07 | 0.75 | 13.7292 | 1.09407 | 13.093 | | 0.12741 | 12.1274 | 0.88149 |

Table 2. Motility parameters from OpenCASA for side-by-side sperm selection comparing SU and MSSP pre and post cryopreservation

|  | **RAW** | **SD** | **Thawed-RAW** | **SD** | **SU** | **SD** | **Thawed-SU** | **SD** | **MSSP** | **SD** | **MSSP** | **SD** |
| --- | --- | --- | --- | --- | --- | --- | --- | --- | --- | --- | --- | --- |
| **VSL** | 49.37 | 17.97377 | 51.04 | 11.99680402 | 60.85 | 11.996804 | 40.56 | 5.583970717 | 61.33 | 12.02289 | 63.01 | 27.33633 |
| **VCL** | 25.37 | 8.472373 | 27.09 | 6.451746398 | 30.21 | 6.4517464 | 19.82 | 3.506737263 | 30.27 | 6.704059 | 28.66 | 12.22573 |
| **VAP** | 34.95 | 4.686537 | 42.10 | 2.145997158 | 36.47 | 2.14599716 | 33.42 | 1.382114472 | 32.66 | 2.585936 | 32.40 | 2.640528 |
| **LIN** | 47.28 | 7.253585 | 54.75 | 2.15648954 | 50.39 | 2.15648954 | 48.92 | 2.198550045 | 50.24 | 3.563692 | 46.70 | 5.000966 |
| **WOB** | 63.96 | 9.309351 | 75.32 | 2.862864874 | 72.29 | 2.86286487 | 69.60 | 1.82834318 | 64.44 | 2.769509 | 71.06 | 6.531136 |
| **STR** | 2.03 | 0.476723 | 2.05 | 0.181158072 | 2.23 | 0.18115807 | 1.66 | 0.070219409 | 1.99 | 0.137518 | 2.01 | 0.656295 |
| **ALH** | 3.33 | 0.712525 | 3.48 | 0.289556792 | 3.42 | 0.28955679 | 4.22 | 0.140560342 | 3.12 | 0.141736 | 2.96 | 0.897917 |
| **BCF** | 132.68 | 59.43637 | 122.00 | 36.29829526 | 154.20 | 36.2982953 | 86.20 | 10.79194478 | 143.56 | 18.28802 | 67.77 | 53.63571 |

Table 3. Sperm assessments before and after SU and Microfluidic Sperm Selection at 15 minutes in oligozoospermic samples (n=6)

| Sperm Metric | Raw Semen | Swim Up | MSSP |
| --- | --- | --- | --- |
| **Concentration (X10^6)** | 8.0 (±9.4) | 1.9 (±0.6) | 1.0 (±0.5) |
| **Progressive Motility (X10^6)** | 39.9 (±15.0) | 85.1 (±6.5) | 96.5 (±2.4) |
| **Sperm Vitality (%)** | 44.5 (±16.4) | 89.1 (±9.0) | 99.0 (±0.9) |
| **DNA fragmentation (%)** | 11.2 (±4.9) | 6.8 (±3.8) | 1.3 (±1.1) |

Supplementary Video 1: Includes a before (left) and after (right) of sperm processed through the motility only MSSP.
